# “Happiness Inventors”: Informing Positive Computing Technologies Through Participatory Design With Children

**DOI:** 10.2196/jmir.6822

**Published:** 2017-01-17

**Authors:** Svetlana Yarosh, Stephen Matthew Schueller

**Affiliations:** ^1^ GroupLens Research Center Department of Computer Science & Engineering University of Minnesota Minneapolis, MN United States; ^2^ Center for Behavioral Intervention Technologies Feinberg School of Medicine Northwestern University Chicago, IL United States

**Keywords:** positive computing, positive psychology, participatory design, cooperative inquiry, children

## Abstract

**Background:**

Positive psychological interventions for children have typically focused on direct adaptations of interventions developed for adults. As the community moves toward designing positive computing technologies to support child well-being, it is important to use a more participatory process that directly engages children’s voices.

**Objective:**

Our objectives were, through a participatory design study, to understand children’s interpretations of positive psychology concepts, as well as their perspectives on technologies that are best suited to enhance their engagement with practice of well-being skills.

**Methods:**

We addressed these questions through a content analysis of 434 design ideas, 51 sketches, and 8 prototype and videos, which emerged from a 14-session cooperative inquiry study with 12 child “happiness inventors.” The study was part of a summer learning camp held at the children’s middle school, which focused on teaching the invention process, teaching well-being skills drawn from positive psychology and related areas (gratitude, mindfulness, and problem solving), and iterating design ideas for technologies to support these skills.

**Results:**

The children’s ideas and prototypes revealed specific facets of how they interpreted gratitude (as thanking, being positive, and doing good things), mindfulness (as externally representing thought and emotions, controlling those thoughts and emotions, getting through unpleasant things, and avoiding forgetting something), and problem solving (as preventing bad decisions, seeking alternative solutions, and not dwelling on unproductive thoughts). This process also revealed that children emphasized particular technologies in their solutions. While desktop or laptop solutions were notably lacking, other ideas were roughly evenly distributed between mobile apps and embodied computing technologies (toys, wearables, etc). We also report on desired functionalities and approaches to engagement in the children’s ideas, such as a notable emphasis on representing and responding to internal states.

**Conclusions:**

Our findings point to promising directions for the design of positive computing technologies targeted at children, with particular emphases on the perspectives, technologies, engagement approaches, and functionalities that appealed to the children in our study. The dual focus of the study on teaching skills while designing technologies is a novel methodology in the design of positive computing technologies intended to increase child well-being.

## Introduction

Two parallel, yet often disconnected, tracks are advancing in the science of technology and well-being. One, stemming from the positive psychology movement, has focused on Web-based positive psychological interventions [1]. This track has mainly translated positive psychological interventions developed offline into Web and mobile versions that could be made widely accessible, able to reach people around the world at the times and places they might most benefit from these interventions. The second track—positive computing technology [2,3]—originates from human-computer interaction and human factors. Positive computing focuses on the design and implementation of technologies that have a beneficial psychological and behavioral impact on the user. Positive computing has often leaned on research in positive psychology to help define what constitutes a beneficial psychological and behavioral impact, borrowing constructs such as subjective well-being [4], or models such as self-determination theory [5] or flourishing [6] to identify and operationalize important targets such as happiness, positive affect, meaning, autonomy, competence, and relatedness. However, with few exceptions, positive computing has rarely focused on translating the principles underlying effective positive psychological interventions into novel technologies developed to promote well-being. As such, interdisciplinary perspectives bringing together positive psychology, human-computer interaction, and human factors are mostly lacking.

This is unfortunate because the pervasiveness of technology affords the potential to reach many more people than can be reached through traditional dissemination of psychological resources. Technology might be especially relevant for children whose time on electronic screens exceeds their time spent either with their parents or in school [7]. However, to successfully design technologies to promote children’s well-being, two types of tailoring are necessary. First, these technologies need to employ design principles that are developed for children to ensure that interaction styles are tailored to children’s interests and capacities (eg, [8]). Second, positive psychological interventions need to align with children’s understanding of the underlying conceptual principles rather than simply adapting language and examples to be age appropriate. In many instances, positive psychological interventions with established efficacy in adults (eg, counting blessings [9]) are simply given to children to evaluate the interventions’ efficacy in new populations (eg, [10]). The alternative approach is to explore what specific positive psychology concepts (eg, gratitude) mean to children and how to promote these concepts within child populations. This approach places children at the center of the process of intervention development rather than viewing them as simply another group for which adaptations need to be made.

In this study, we took a participatory design approach to understanding children’s perspectives on positive psychology concepts such as gratitude, mindfulness, and problem solving and worked from these perspectives to iterate ideas for positive computing technologies. Through a 14-session participatory design study with 12 children, we elicited children’s interpretations of these concepts and positive computing technology designs to answer the following research questions (RQs). RQ1: What do children’s “happiness inventions” reveal about their perspectives on happiness and positive psychology concepts? RQ2: What kinds of positive computing technologies and approaches are emphasized in children’s designs? RQ3: How can positive computing technology designs targeted at children better match their mental models and priorities?

To position our work, we begin this paper by contextualizing both our methodological approach of participatory design and the specific positive psychology skills and concepts emphasized in our 14-session study. Next, we describe the specific operationalization of participatory design we enacted in this investigation. We discuss the design ideas generated and developed by the children in this study to address RQ1 and RQ2. Finally, we return to RQ3 with implications for design in this context.

### Participatory Design

The basic principle of the participatory design approach is that people who are affected by the introduction of a new technology have the right to participate in the creation of this technology. Participatory design has become an important approach in human-computer interaction as a set of theories, practices, and studies related to end users as full participants in activities leading to the creation of technologies [11]. In the late 1990s, this approach was adapted to support intergenerational design partnerships with children, terming this adaptation the cooperative inquiry method [12]. The method has been used in many projects—for example, designing a children’s digital library [13]. It provides benefits in terms of both leading to more creative and better-situated final outcomes [14] and increasing the agency of and empowering the child partners involved in the design process [15]. Our goal reflected the priorities of cooperative inquiry and participatory design—to create well-contextualized digital artifacts to support teaching well-being skills to children. As in other cooperative inquiries, we were also interested in providing benefits to study participants in terms of both fostering agency [15] and teaching specific, actionable, and useful skills (in this case, design thinking; science, technology, engineering, and mathematics education; and positive psychological skills to promote well-being) to participants.

### Technology Design for Well-Being and Resilience

Positive computing technology is a well-explored and growing priority in both positive psychology and human-computer interaction. Some particularly noteworthy examples of projects are a mobile intervention to help capture and reflect on positive occurrences and thoughts [16]; a context-sensitive mobile app designed to promote gratitude [17]; a sensor-based interface for supporting meditation practices [18]; and conceptual physical computing designs to help promote mindfulness [19]. Although these projects are generally viewed as effective, sustained engagement with such programs is a considerable barrier (eg, [20]). These barriers may be amplified when technologies or interventions that are designed for adults are adapted and deployed with children. As we seek to include children in well-established positive psychology practices such as gratitude, mindfulness, and problem solving, we must review and engage with previous work to understand the goals, potential benefits, and potential challenges of such interventions. We briefly review each of the three practices below.

#### Gratitude

Promoting gratitude in children is a key interest of researchers and educators. Gratitude refers to the disposition to recognize good things and an appreciation for receiving these things [9]. Gratitude is a social emotion that serves several functions, including facilitating and strengthening relationships [21] and promoting subsequent prosocial behavior [22]. Mounting evidence suggests that interventions can effectively increase gratitude and subsequently improve well-being in adults and children [23-25]. Child-targeted modifications of common gratitude interventions (eg, counting one’s blessing [9], gratitude visits [26,27]) include conducting the intervention in a relational context [28] and providing it to children in sixth- and seventh-grade classrooms [10,23]. From this and other previous work, gratitude appears to be a beneficial and teachable skill for children; thus, we chose it as one of the concepts we fostered in this study.

#### Mindfulness

Mindfulness refers to a skill to attend to one’s present environment in a receptive and nonjudgmental fashion [29]. Mindfulness skills have been linked with improvement in stress management, mood, and behavior [30-32], and consistent mindfulness practice has been linked to beneficial structural changes in the brain [33]. Mindfulness interventions are becoming increasingly popular in school-based settings, with research reports demonstrating their effectiveness and detailing their implementation [34-38]. Despite the benefits of these interventions, their successful implementation in school-based settings faces several barriers. These issues include communication among facilitators, acceptance by school administrators, the necessary space, time, and resources to conduct instruction, and perceptions of mindfulness and related practices as “primarily an activity of white privileged females” [38] (pg 281). These barriers emphasize the importance of working alongside stakeholders to build relationships and the need to frame mindfulness practices appropriately for those for whom the intervention is intended. Although operationalizing mindfulness for specific populations remains an open question, mindfulness principles have been successfully taught to children and have broad benefits that contribute to their well-being. As such, mindfulness was the second concept we selected as a focus in this study.

#### Problem-Solving and Cognitive Skills Training

Problem-solving skills training is both a standalone treatment [39,40] and a major component of cognitive behavioral therapy (which is one of the most widely researched and validated treatments for a host of social and emotional issues for children [41,42]). Increasingly, schools have recognized the value of teaching these problem-solving skills to boost well-being and resilience before social and behavioral issues occur (eg, [43,44]). One of the most widely researched resilience programs for children is the Penn Resiliency Program, which was originally designed as a school-based program but has been evaluated in other settings, including primary care clinics [45] and juvenile detention centers [46]. Several studies have evaluated its effectiveness to reduce depressive symptoms and have found reliable but small benefits [47]. These skills are relevant and teachable to children even at young ages, and supporting these skills can contribute to personal, interpersonal, and academic success, leading us to select problem solving as the third and final emphasized concept in this investigation.

### Positive Psychology in the Classroom

Ours was not the first project to attempt to bring positive psychology skills into the classroom. Previous efforts have been based on the notion that well-being skills are fundamental (yet often overlooked) goals of education [48]. Furthermore, well-being is teachable through specific skills that can complement rather than constitute academic learning objectives. Lastly, increasing well-being can benefit education through promoting learning. Positive moods have been found to broaden attention [49], increase creativity [50], and promote more holistic [51] and analytic thinking [52]. As such, positive psychological skills have been taught in educational settings either as adjunctive programs or integrated more tightly with traditional educational lessons. A complete review of all such programs is beyond the scope of this paper, as teaching well-being skills was only one aspect of our larger program, serving as the context for our participatory design process. Nevertheless, we discuss a few programs that inspired our selection of well-being strategies and offer potential paradigms in which positive psychological interventions or positive computing technologies inspired by our program could be integrated.

As previously mentioned, the Penn Resiliency Program was designed for school-based administration and has been successfully disseminated to different populations by tailoring several aspects of its delivery. A meta-analysis of 17 controlled evaluations of Penn Resiliency Program found reliable but small benefits in terms of reduction of depressive symptoms [47]. The review also found that programs led by members of the initial Penn Resiliency Program research team experienced greater benefits than those led by community providers. This suggests that, although such programs can be beneficial, issues of successful dissemination and training might affect their effectiveness when scaling. Positive computing technologies serving as supports to the intervention could potentially increase the scalability of such efforts. Other efforts have attempted to intertwine positive psychology principles directly into education. As an early example, the Positive Psychology Program evaluated providing language arts education that aimed to help students identify their character strengths and increase their use of these character strengths in their lives [48]. These programs were foundational for the Geelong Grammar School model for positive education, which integrates well-being skills deeply into classroom education through a “live it, teach it, embed it” philosophy [53]. These efforts demonstrated that teaching well-being can be beneficial and relevant to the educational context, if scaled effectively. Positive computing technologies could go a long way in making these efforts more scalable and sustainable.

## Methods

We conducted 14 cooperative inquiry sessions lasting 90 minutes each with 12 sixth and seventh graders. During these sessions, we contextualized the participatory design process by practicing specific positive psychology skills, we conducted ideation sessions, and we prototyped and documented selected ideas that emerged through this process. Multimedia Appendix 1 includes a more thorough description of these skills organized by topic areas (gratitude, mindfulness, and problem solving).

### Setting

Participants were recruited from a summer learning program offered by a local youth development agency (Youth & Opportunity United [Y.O.U.] Program [54]) in a suburban middle school outside of Chicago, Illinois, USA. The summer learning program’s theme was “Technology and its Impact on Society,” which was an 8-week, 4.5-hours-per-day program enrolling approximately 40 middle-school students. Students enrolled in this program were allowed to choose between one of three elective projects, one of which was the Happiness Inventors study we led. The students were informed that the Happiness Inventors elective was a research project and that informed consent would be required to participate in this elective. Participants in elective projects met twice a week (Mondays and Wednesdays) for 1.5 hours after lunch and free recreation, and before debriefing and snack. No elective session was held on the first day of the summer learning program and one Monday was a national holiday. Thus, our study consisted of 14 total sessions. As attendance in the summer learning program was not mandatory, the number of participants at each session differed considerably from day to day. We did not formally take attendance during each session but constructed attendance data based on evidence of participants’ activities (eg, writing in their invention notebooks). Table 1 documents individual attendance by session.

**Table 1:** Demographics and attendance of the 12 children taking part in the Happiness Inventors elective.

Participant number	Age (years)	Sex	Race/ethnicity	Session number													
				1	2	3	4	5	6	7	8	9	10	11	12	13	14
1	12	Male	White	X		X	X	X		X		X	X	X	X		
2	11	Female	African American	X	X	X	X	X	X	X	X	X	X	X	X	X	X
3	12	Male	White	X	X		X	X	X	X		X	X	X	X		
4	11	Male	White	X	X		X	X	X	X		X		X		X	X
5	N/A^a^	Male	Hispanic or Latino	X	X			X	X			X	X	X		X	
6	11	Male	African American		X	X						X	X	X	X	X	X
7	N/A	Female	African American	X	X	X	X	X			X	X	X	X	X		
8	N/A	Male	African American		X	X		X	X	X		X	X	X		X	
9	12	Female	White	X	X		X	X	X	X	X	X	X	X	X	X	X
10	N/A	Female	Hispanic or Latino		X			X	X	X	X	X	X	X	X	X	
11	N/A	Male	Hispanic or Latino	X	X	X		X		X		X			X	X	
12	12	Female	White	X	X	X	X	X	X	X	X	X	X	X	X	X	X

^a^N/A: not available.

### Participants and Recruitment

A total of 12 children participated in our study, along with 2 researchers (SY and SMS) and 1 behavioral aid provided by the summer learning program. Complete demographic data were available from 8 children (the other 4 missed classes where data were collected and contributed only partial data). Table 1 displays demographic data for each participant. The children were predominantly seventh graders (5/7, 71%), with a mean age of 11.57 (SD 0.54) years. The group consisted of more boys (7/12, 58%) than girls (5/12, 42%) and was ethnically diverse, with 42% (5/12) non-Hispanic or Latino white, 33% (4/12) African American, and 25% (3/12) Hispanic or Latino.

All Y.O.U. summer program participants were provided with the opportunity to choose to take part in this Happiness Inventors study. Those who expressed interest after an introductory session filled out assent forms and were given parental consent forms to take home and return. Any child could choose to stop participating in the study at any time (by switching to one of the other elective sessions). Additionally, parents could elect to pull their child from any given session (eg, if the family was going on vacation that week). The institutional review boards at both the University of Minnesota, Minneapolis, MN, USA, and Northwestern University, Chicago, IL, USA, approved this project.

### Procedure

In this study, we worked with children to understand and design positive computing technologies. As in previous work with children, we adapted a cooperative inquiry approach to participatory design [55], enlisting the children in the study both as inventors and as fellow investigators. This cooperative inquiry took place over the course of an 8-week summer program, with two 90-minute sessions each week. The goals and structure of each week were as follows.

#### Orientation and Introduction

All camp children were introduced to the investigators and given the opportunity to join the study. As the group first came together, we jointly created and signed a charter to guide our collaboration on the project (including rules such as “respect ideas in how you give feedback”).

#### How to Be an Inventor

A computer scientist (first author, SY) with years of experience being an inventor introduced the children to the process of inventing (including the importance of formative work, ideation, and prototyping). We also introduced invention notebooks as a common industry practice for documenting patentable ideas. The goal of these workshops was to position the ideation, documentation, and low-fidelity prototyping processes as authentic practices of real inventors.

#### Happiness and Gratitude

A clinical psychologist (second author, SMS) with expertise in positive psychology introduced the children to the idea of happiness as a practice. Gratitude was the first happiness skill introduced to children through age-appropriate exercises (see Multimedia Appendix 1 for more details about these exercises). This session provided them with the opportunity to reflect on the fairly abstract concepts of happiness and gratitude and become investigators of their own experience as they practiced the taught skills.

#### Mindfulness and Problem Solving

The clinical psychologist continued teaching positive psychology skills, focusing on mindfulness and problem solving through age-appropriate exercises (see Multimedia Appendix 1 for more details about these exercises). This session provided the children with the opportunity to reflect on these fairly abstract concepts and become investigators of their own experience as they practiced these skills.

#### Technical Possibilities Workshop

Two experts in mobile app development and embodied computing (eg, wearable technologies) each led 1 session with the children, describing common approaches to prototyping technology in their respective fields. Technologies covered included low-fidelity prototyping, the *Prototyping on Paper* app (Woomoo Inc, Taipei, Taiwan), littleBits electrical circuit kits (littleBits Electronics Inc, New York, NY, USA), and the Oculus Rift virtual reality head-mounted display (Oculus VR, LLC, Menlo Park, CA, USA). The goal of these workshops was to broaden the children’s perspectives on what constitutes technology, in order to inform the ideation stage.

#### Gratitude Ideation

The children used the IDEO ideation approach [56] in small groups (approximately 4 children depending on attendance and 1 adult per group) to generate 180 ideas for technology that could help other children practice gratitude. They reflected on the best ideas in their invention notebooks, voted on clusters of ideas, regrouped by favorite cluster, and created detailed sketches and videos of their final ideas. (Multimedia Appendix 2 documents all ideas.)

#### Mindfulness Ideation

The children used the same approach to generate 152 ideas for technology that could help other children practice mindfulness. They reflected on the best ideas in their invention notebooks, voted on clusters of ideas, regrouped by favorite cluster, and created detailed sketches, prototypes, and videos of their final ideas. (Multimedia Appendix 2 documents all ideas.)

#### Problem-Solving Ideation

The children used the same approach to generate 102 ideas for technology that could help other children practice problem solving. They reflected on the best ideas in their invention notebooks, voted on clusters of ideas, regrouped by favorite cluster, and created detailed sketches, prototypes, and videos of their final ideas. On the last day of the study, we also reserved time for final reflection and to view the videos created by the children throughout the summer. (Multimedia Appendix 2 documents all ideas.)

We took an action research approach (eg, [57]) to iteratively structure the workshops, keeping in mind the overarching goals of equalizing power between the children and the researchers, increasing the children’s acceptance of the project, and adjusting specific plans based on the attendance and participation on any given day. For example, our original plan was to structure each ideation workshop in two parts, where children first focused on designing apps and then on designing other technologies. But, during the first workshop (focused on gratitude), we found that this artificial division was frustrating for the children, and they were generally less excited about focusing on app ideas. In subsequent workshops, we did not prompt this separation. As another example, the action research approach led us to change our video data collection process. During the first ideation workshop, we set up webcams to record each group’s progress. We found these to be distracting for the children and that these amplified the power differential between them (as data sources) and us (as data collectors). In subsequent sessions, we instead asked the children to document their own ideas and process using the cameras. Enlisting the children as the directors of their own self-documentaries increased their agency and willingness to participate (although at the expense of objective data quality).

We collected data from many sources throughout this process. The primary sources used in these analyses were the transcripts of the ideas generated by the children, the reflections and sketches in the children’s invention notebooks, and the transcripts of the video documentation collected by them during their invention process.

### Qualitative Content Analysis

To support qualitative analysis, we converted all data from the study into a textual format as follows: (1) transcribing all (434 total) ideas from the ideation sessions, (2) transcribing all written notes and describing all (51 total) drawings from the children’s invention notebooks, (3) transcribing and describing in words all (8 total) video-documented ideas and prototypes from the ideation workshops.

We conducted a data-driven inductive thematic analysis, characterized by the generation and constant comparison of open codes [58]. The first author (SY) conducted the open coding by reading through all of the transcribed data and adding one or more short descriptive phrases to label each idea. No clustering was attempted at this stage. SY then read through the open codes, added memos, and initiated discussions with the second author (SMS) to begin noting and articulating interesting themes. Based on the open codes, memos, and discussions, the first author (SY) and a student apprentice applied affinity mapping to cluster the resulting set of open codes and memos to identify patterns and overarching themes in the data. The first author described the resulting set of 47 clustered codes as a codebook, specifying concrete inclusion and exclusion criteria for applying a particular code. To ensure that this codebook was clear and the codes could be consistently applied, the 2 coders (authors SY and SMS) independently applied the codebook to categorize a randomly selected set of ideas (43/434, 9.9% of the full set of ideas, where 1 or more of the 47 codes could be applied to each idea). The 2 coders achieved strong agreement (Cohen kappa=.80) as calculated using the Cohen kappa test of nominal data agreement by 2 coders [59]. The coders discussed all cases of disagreement until reaching consensus, and the specific points of that discussion were encoded as modifications of the codebook (eg, the “robot” code should be applied to any idea that includes a “drone”). The first author (SY) then applied the modified codebook to coding the remaining 391 ideas, 51 notebook entries, and 8 videos. We present the major results of this process and provide specific examples of each code in the Results section.

## Results

**Figure 1:**
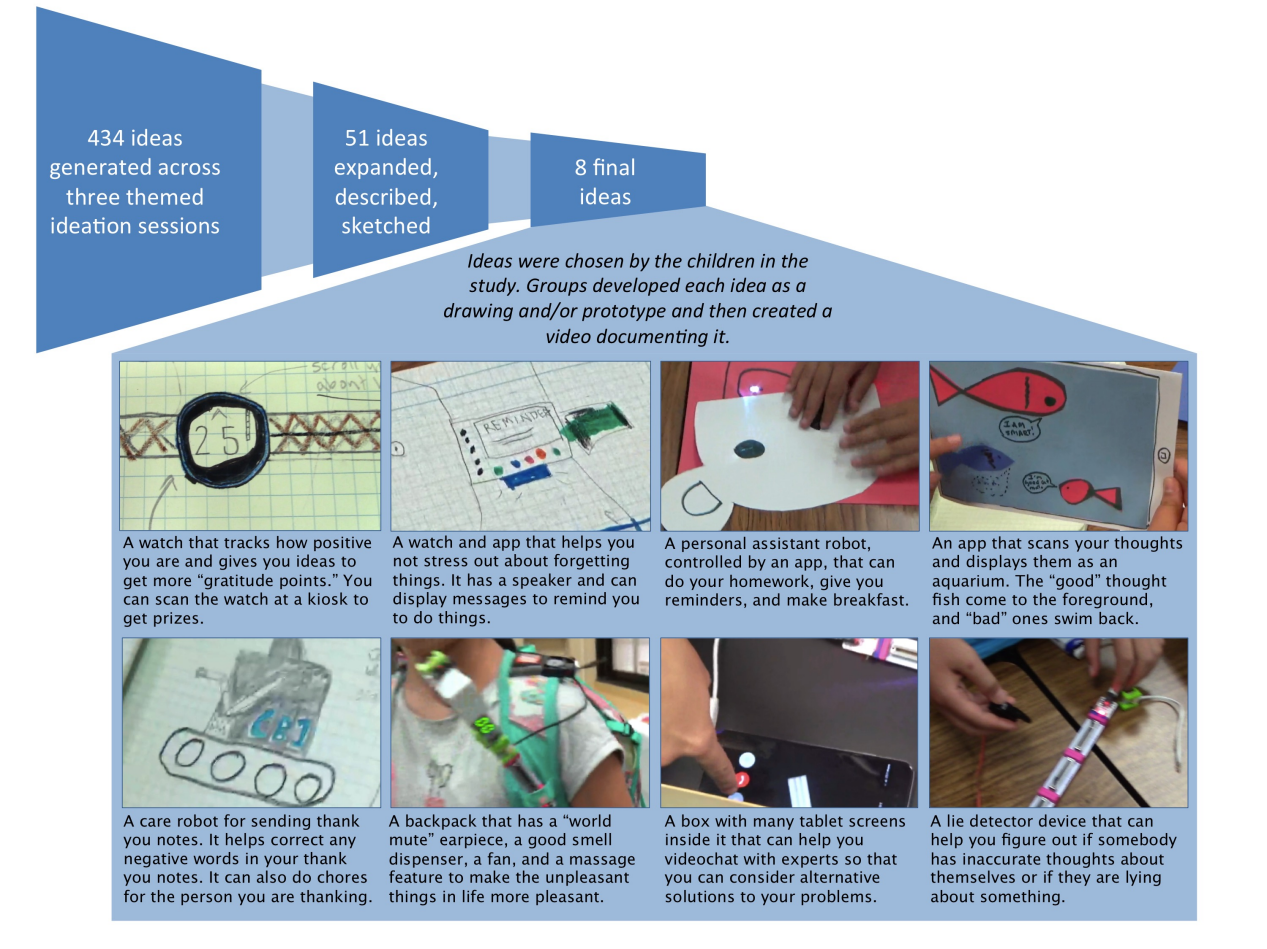
Summary of the ideation and idea selection process showing a brief description and a still image captured from each video of each of the final 8 ideas chosen by the children for their prototyping and video documentation.

In this section, we provide empirical data to address the first 2 research questions: how children interpret positive psychology concepts and which technological approaches are emphasized in their designs. In the Discussion, we return to the third research question of how to best design and target positive computing technologies for children. Figure 1 describes the quantitative characteristics of the ideation, selection, and documental process, along with images and short descriptions of the final design ideas that the children chose to document as low-fidelity prototypes and videos.

### RQ1: What Do Children’s Happiness Inventions Reveal About Their Perspectives on Happiness and Positive Psychology?

We coded the ideas that the children generated (434 ideas total), documented in notebooks (51 designs), and developed as prototypes and videos (8 total) (Figure 1) for specific interpretations of each of the positive psychology skills covered in the cooperative inquiry sessions. Multimedia Appendix 2 provides the complete list of ideas.

#### Interpretations of Gratitude

**Figure 2:**
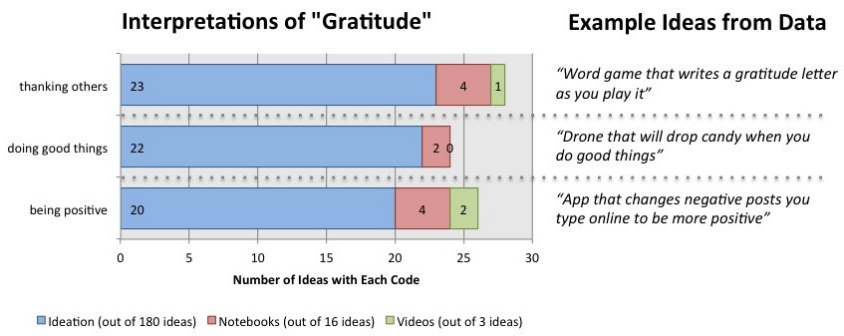
Prevalence of each of the 3 codes for interpretation of “gratitude” observed in the children’s ideas, documentation, and videos.

During the weeks focused on the concept of gratitude, the children generated 180 ideas, 16 notebook sketches, and 3 videos describing ideas for technologies that would help children practice gratitude skills as they interpreted them. Not all of the ideas expressed a specific interpretation, as the IDEO process specifies deferring judgment at the ideation stage (ie, many of the ideas were irrelevant to gratitude). We coded relevant ideas for implicitly or explicitly expressed interpretations of gratitude, finding a relatively equal split between three concepts (Figure 2). The most common interpretation was that practicing gratitude is about thanking others. Many of the ideas in this category focused on writing thank-you notes, making gifts, etc, for others. The next most common interpretation focused on generally remaining positive in life. Ideas included devices or apps that enforced or rewarded positive thinking (or punished negativity). Finally, another interpretation focused on gratitude as enacted by “doing good things” (typically for others), and many ideas in this section focused on encouraging people to “do good things” and engaging with people who need help (eg, donating to charity, helping a friend who is not feeling well).

#### Interpretations of Mindfulness

**Figure 3:**
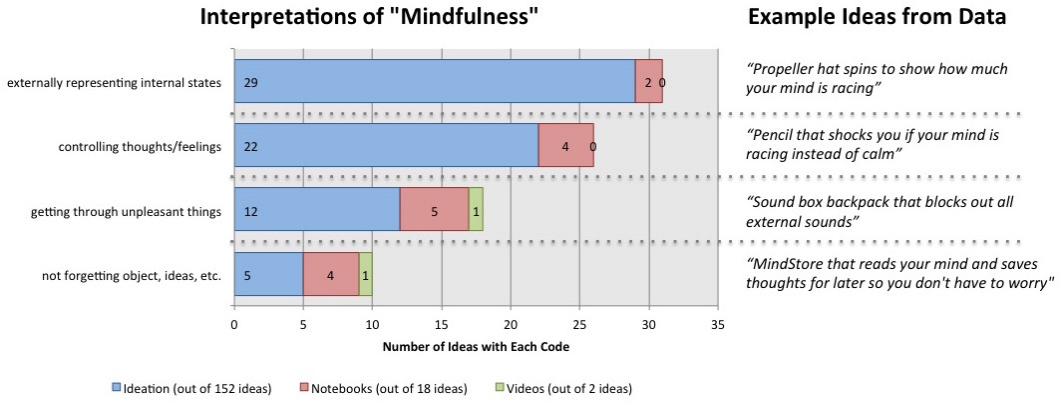
Prevalence of each of the 4 codes for interpretation of “mindfulness” observed in the children’s ideas, documentation, and videos.

During the weeks focused on the concept of mindfulness, the children in the study generated 152 ideas, 18 notebook sketches, and 2 videos describing ideas for technologies that would help children practice mindfulness skills as they interpreted them. Not all of the ideas were relevant to the prompt or expressed a specific interpretation. However, it is worth noting that the relative prevalence of relevant ideas increased from 36.1% (65/180) in gratitude sessions to 44.7% (68/152) in mindfulness sessions. We coded relevant ideas for implicitly or explicitly expressed interpretations of mindfulness as a concept, identifying four major themes (Figure 3). The most common interpretation was that the best way to practice mindfulness is by externally representing internal states. Many of the ideas in this category focused on creating physical and visible manifestations or representations of emotions and thoughts. The next most common interpretation focused on controlling thoughts and feelings. Ideas included erasing unwanted thoughts or transitioning from a “bad” emotional state (mind racing, feeling sad) to a “good” emotional state (calm, feeling happy). The next class of ideas focused on mindfulness as a way of getting through unpleasant external situations. Many of the ideas in this class focused on avoiding or removing oneself from unpleasant situations and controlling the sensory aspects of one’s environment (eg, sound, smell). The final interpretation saw “being mindful” as the opposite of being “absentminded” and operationalized this idea as preventing a person from forgetting an object or idea. Many of the ideas in this category focused on saving ideas and reminding one to take specific actions. While the last two interpretations were not as strongly represented in the ideation process as the first two, they seemed to get more vetting from the children by being documented as sketches, prototypes, and videos.

#### Interpretations of Problem Solving

**Figure 4:**
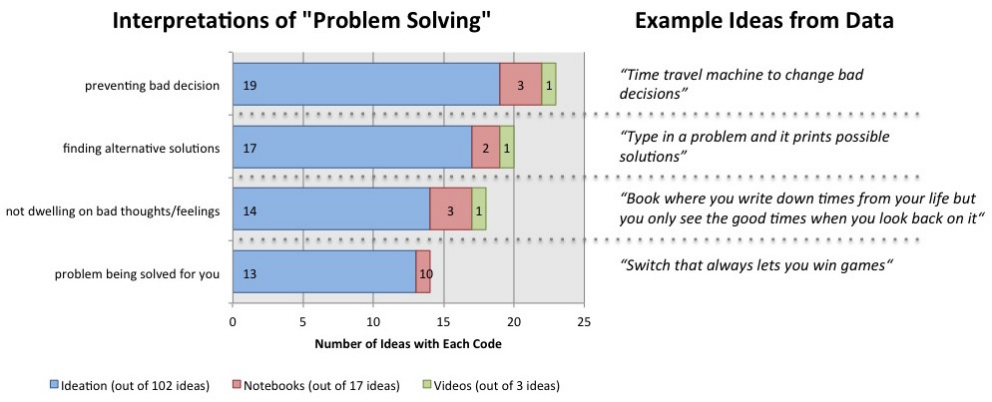
Prevalence of each of the 4 codes for interpretation of “problem solving” observed in the children’s ideas, documentation, and videos.

During the weeks focused on the concept of problem solving, the children generated 102 ideas, 17 notebook sketches, and 3 videos describing ideas for technologies that would help children practice problem-solving skills as they interpreted them. We saw a relative increase in the number of relevant ideas that emerged from the ideation, from 36.1% (65/180) in gratitude sessions and 44.7% (68/152) in mindfulness sessions to 61.7% (63/102) in problem-solving sessions. We coded relevant ideas for implicitly or explicitly expressed interpretations of problem solving as a concept, identifying four major themes (Figure 4). The most common interpretation was that the best way to practice problem solving is by preventing a person from making a bad decision. Many of the ideas in this category focused on providing additional information about the issue at hand, getting more time to think about a decision, and finding ways to control the damage when bad decisions were made. The next most common interpretation focused on finding alternative solutions. Ideas included generating lists of possible solutions and turning to others to identify new ways of looking at a problem. The third category focused on problem solving as preventing dwelling on ideas or feelings that may be unproductive. Many of the ideas in this class focused on erasing specific thoughts or feeling or getting others to help correct inaccurate thoughts. The final interpretation of problem solving focused simply on the solution itself, typically generated automatically for the user by a device or person. These ideas focused on devices that solved specific situations in the children’s lives that they saw as problems, such as doing homework, completing chores, losing at games, and getting bullied. This is a somewhat naïve interpretation of problem solving, but it is important to note that the vetting process of documenting the best ideas as sketches, prototypes, and videos largely removed this interpretation from the set. Children were able to see this interpretation of problem solving as naïve and gravitated toward more productive ideas.

### RQ2: What Kinds of Positive Computing Technologies and Approaches Are Emphasized in Children’s Designs?

We also took note of the specific aspects of the technology solutions generated by the children. We divide this discussion into three major aspects of solutions: technology employed, functionalities described, and approach used to engage the user. These reflect a post hoc clustering of the codes observed in the data, rather than any specific prompts given to the children during the design process.

#### Featured Technologies

**Figure 5:**
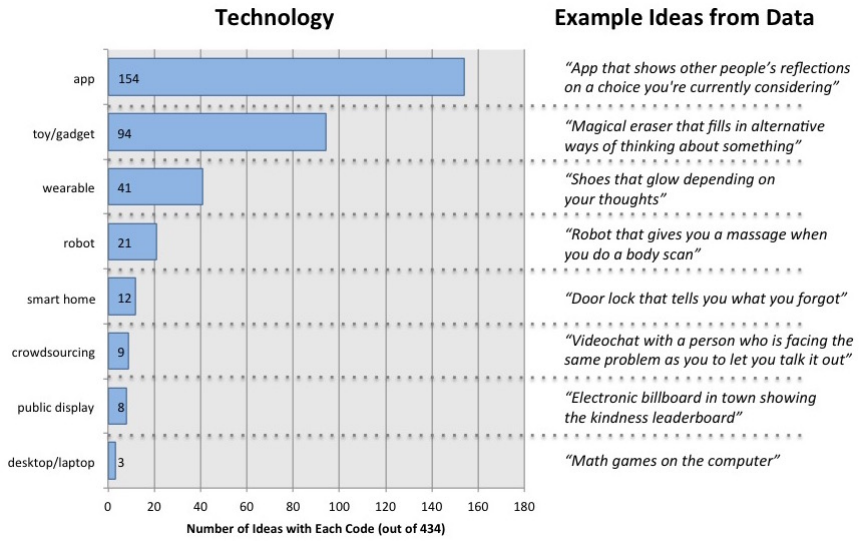
Prevalence and examples of each of the 8 codes for technological solutions observed across 3 ideation workshops.

We categorized ideas generated throughout the 3 ideation sessions based on the technological solution featured (Figure 5). While the reader should be familiar with technologies such as apps (154 ideas), toys (94), and robots (21), a few of the other terms may need to be defined (none of these words were used explicitly by the children, but they are the industry terms for the ideas described):

- Wearable (41 ideas): an on-the-body technology worn as an accessory (eg, watch, jewelry, glasses) or as apparel (eg, shirt, shoes).
- Smart home (12 ideas): digital intelligence embedded in home infrastructure, appliances, or furniture.
- Crowdsourcing (9 ideas): leveraging technology to structure an interaction with strangers who provide a service or information.
- Public display (8 ideas): a public device for distributing content, such as a billboard, kiosk, or information panel.

The most common category of technology included in the children’s ideas was a phone or tablet app, although it is important to note that roughly half of these (71) came from the first workshop, where we specifically asked them to come up with a total of 90 app ideas (and then 90 physical ideas). In subsequent sessions, where we did not enforce any technology-specific breakdown of ideas, children gravitated to embodied technologies (toy/gadget, smart home, wearable, robot, public display) to a greater extent. In the mindfulness ideation workshop, these 5 categories jointly accounted for 65 ideas, while apps were mentioned in 47. In the problem-solving workshop, these 5 categories accounted for 45 ideas, while apps were mentioned in 36. An interesting note is that conventional, on-the-desktop technology ideas were almost completely absent from the children’s ideation. Only 3 ideas out of the 434 mentioned computers, laptops, or websites. This may highlight the importance of a mobile-first approach in designing Web-based interventions for children.

Additionally, throughout the ideation process, many ideas did not match specific technologies. For example, a total of 34 (out of 434) ideas were coded as “No Technology” (eg, “Play with a pet”) and a total of 19 (out of 434) ideas were coded as “Magic” (eg, “Something that grants you 16 wishes”). The frequency of these types of ideas decreased through the duration of the study, and none of these ideas were documented in invention notebooks or videos.

#### Common Solution Features and Functionalities

**Figure 6:**
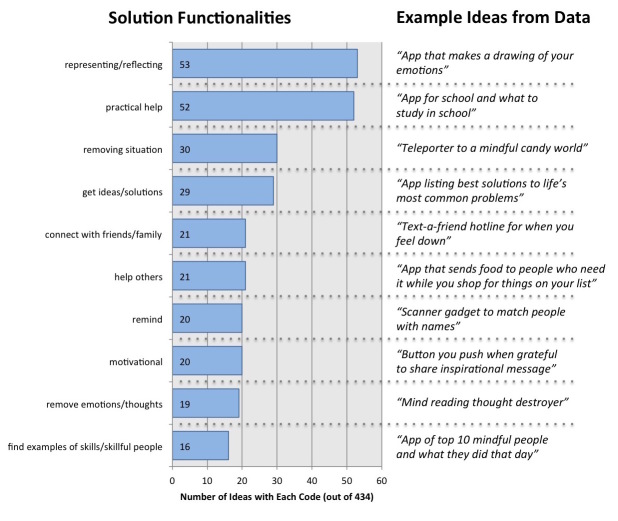
Prevalence and examples of each of the 10 codes for solution functions observed across 3 ideation workshops.

Through our coding process, we also noted that certain functionalities kept reappearing in children’s ideas across all 3 ideation workshops (Figure 6). A few of these are particularly interesting. The most common functionality presented in the technology solutions was an opportunity to represent and reflect internal states. Particularly, many of the ideas focused on representing ideas, feelings, and thoughts as physical or digital objects to see and manipulate at will. Related to this idea, several of the solutions focused on the ability to remove feelings (eg, sad, tired) or thoughts (eg, feeling insecure, having negative thoughts about someone) at will. It is also telling how frequently solutions included connecting with others. The children designed technologies to connect with friends and family, to help others in their communities, and to find examples of people modeling happiness skills.

However, not all ideas focused on happiness as an internal process. Many of the children manifested the belief that happiness is largely due to the external environment. Many of the ideas addressed the specific *causes* of stress in their lives, such as the need for practical help (eg, homework, chores) and frustrations with forgetting something important (eg, object, activity, idea). Many of the other solutions focused on removing yourself or others from a problematic situation rather than dealing with the situation itself. These ideas included teleporters, “disappearing machines,” “day restarters,” invisibility cloaks, and “world pause” buttons.

#### Strategies for Sustained User Engagement

**Figure 7:**
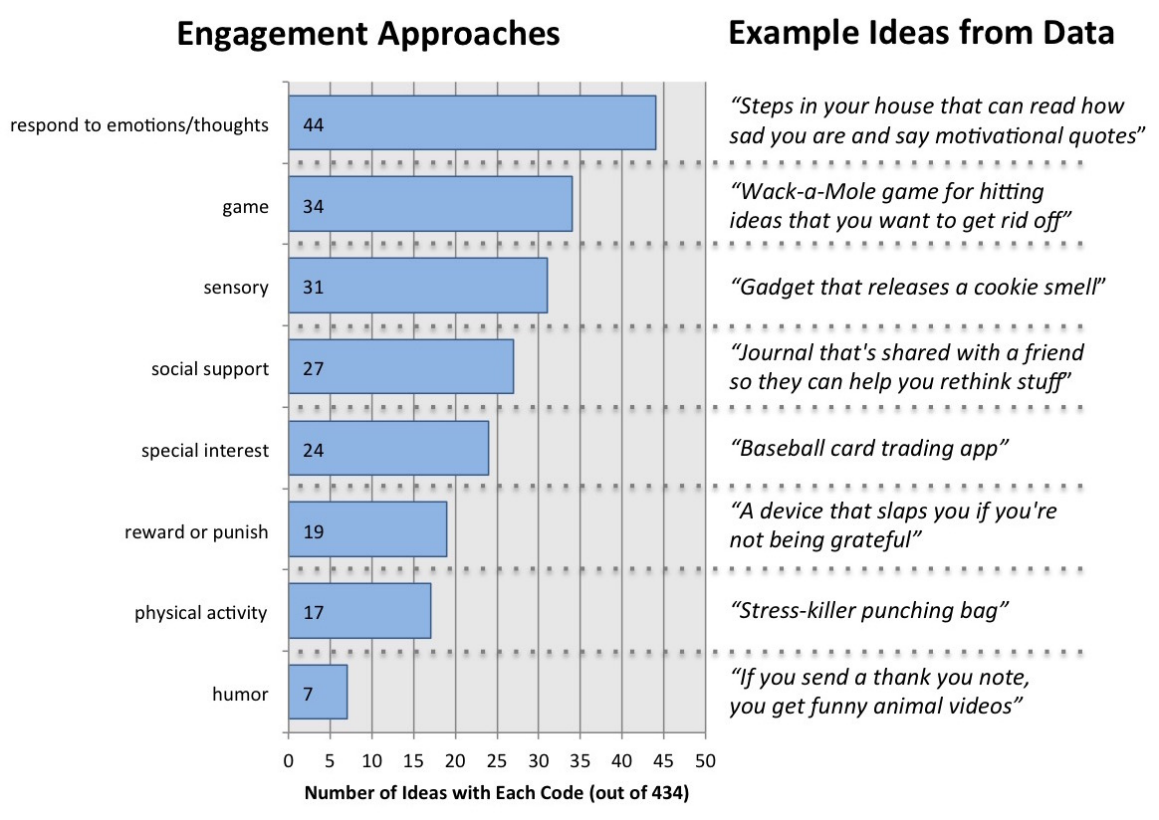
Prevalence and examples of each of the 8 codes for engagement approaches observed across 3 ideation workshops.

Children in this study seemed to have an intuitive understanding that designing a technology to practice a specific happiness skill requires consideration of *why* somebody would engage and continue engaging with a particular device or app. A total of 8 of the codes highlighted specific approaches for engaging the user (Figure 7). It is not surprising to see games as one of the top strategies on this list. However, it may be more surprising that games were just one of the ideas suggested and not the most prevalent one. Rather than seeking to be entertained with games, the children focused on solutions that could understand and engage directly with their emotions and thoughts. In addition to playfulness and responsiveness, the children emphasized solutions that engaged with all the senses, allowed them to seek out social support, and connected with existing special interests that they had (eg, the Chicago Bulls basketball team). Early in the design process, they also frequently focused on the idea of engagement through rewarding skill practice or punishing lack of skill practice, but these ideas seemed to garner less favor as the sessions continued (only 8 of the 19 ideas that featured rewards or punishment came from the last 2 ideation sessions). It seemed that children favored more nuanced interpretations of engagement as they had more experience with ideation.

## Discussion

To our knowledge, this is the first study to employ a participatory design approach to the development of positive computing technologies for children, building on principles of positive psychological interventions. We reflect on this process and provide implications for the design of positive technologies.

### Principal Results

We generated 434 ideas, 51 sketches, and 8 videos of potential positive technologies through a 14-session cooperative inquiry process with 12 children. These ideas and prototypes revealed specific facets of how children interpreted gratitude (as thanking, being positive, and doing “good things”), mindfulness (as externally representing internal states, controlling those states, getting through unpleasant things, and avoiding forgetting something), and problem solving (as preventing bad decisions, seeking alternative solutions, and not dwelling on unproductive thoughts). This process also revealed the particular technologies that were emphasized by the children in their solutions. While there was a notable lack of desktop and laptop solutions, other ideas were roughly evenly distributed between apps and embodied computing (toys, wearables, etc) ideas. Finally, we were able to understand both the desired functionalities and approaches to engagement in the children’s ideas, with a notable emphasis on representing and responding to internal states. Our work points to new promising directions in the design of positive technologies with and for children.

### Methodological Reflections

One of the methodological factors underscored through this participatory design process was the importance of ongoing engagement with and iteration of ideas. As the children in this study practiced ideation, they were able to increase the relative number of relevant ideas generated: from 36.1% (65/180) in the first workshop, to 44.7% (68/152) in the second one, to 61.7% (63/102) in the final ideation session. Additionally, we observed that allowing the children multiple sessions to gain some distance from their ideas, document their favorites, and reflect served as a vetting process that favored more nuanced interpretations of certain concepts. For example, the children largely ruled out the naïve “problem solving is having problems solved for you” interpretation, which appeared in ideation but not in subsequent stages. The opportunity to reflect also focused the design process, weeding out many ideas that were not relevant to positive technology and ideas that focused on “magical” solutions. These benefits from ongoing engagement and iteration would not have been possible had we conducted a single focus group or multiple focus groups with different children as a way of eliciting ideas. In fact, our 14-session format is unique among work in this field, yet helped build a deep engagement with the children as well as the youth organization. This sustained engagement is not without challenges, such as keeping the children interested over time and managing evolving relationships, but the benefits realized in terms of ideas produced would likely not be possible otherwise.

### Comparison With Prior Work

We expanded on past work that applied user-centered practices to the development of positive psychological interventions [60] and positive technologies [61] by engaging in cooperative inquiry with children around positive technologies. Our work is novel in that we engaged in participatory design around positive psychology skills drawn from empirically validated positive psychological interventions (eg, gratitude, mindfulness, and problem solving) and we explicitly taught these skills as part of the cooperative inquiry investigation. While this allowed us to benefit from the previous investigations in positive psychology, our unconstrained ideation process also supported a broader perspective than simply creating digital versions of existing interventions (a common approach in positive technology development [62]). For example, studies in this domain have tended to replicate (eg, [63]) or create new versions of (eg, [64]) Seligman and colleagues’ seminal study [27], which evaluated a gratitude visit, three good things, optimism, and two signature strengths exercises disseminated through a website. Our findings have the strongest bearing on and relevance to the design of positive psychological interventions by revealing several technologies (eg, embodied computing), engagement approaches (eg, responding to internal states, sensory engagement, humor), and functionalities (eg, representing internal states, crowdsourcing solutions and examples) that may be promising in designing positive interventions for children (we discuss the implications of this in more detail below).

Some of our findings have relevance not just to positive technology, but also to positive psychological interventions more generally, incorporating children’s views on happiness, gratitude, mindfulness, and problem solving. First, it is worth noting that several design ideas (7.8%, 34/434) were not related to technology whatsoever. Children continued to think of happiness and happiness strategies more broadly even with our explicit focus on technology. Of their design ideas, many had some aspect both of enhancing positive aspects of the children’s experience and for removing negative or problematic aspects of it. This is quite different from most positive psychological interventions, which tend to promote happiness through didactic instruction in well-being skills (eg, [48,65]). Second, this “removing negative” experiences pathway to happiness is inconsistent with most conceptual thinking in positive psychology about what is unique about positive psychological interventions compared with other clinical approaches [66-68]. However, external supports and contingencies might align more with children’s mental models and capacities, as concrete examples are often necessary to help support cognitive and other regulatory processes (eg, [69]). As such, positive psychological interventions that use physical artifacts to embody abstract concepts or provide additional support may be particularly beneficial for children (eg, [60]).

### Implications for Design of Positive Computing Technologies for Children

In this section, we propose some directions to address our third research question of how positive technology designs targeted at children can better match their mental models and priorities.

Children’s interpretations of positive psychology concepts such as gratitude, mindfulness, and problem solving may not always match adult interpretations and perspectives of these concepts. For example, some of the children interpreted mindfulness as “not forgetting ideas or objects” or problem solving as “having problems solved for you.” Additionally, many children’s interpretations of happiness across all three concepts revolved around external influences on happiness, such as getting practical help (eg, with homework) or avoiding unpleasant situations. These may not be typical concepts within positive psychology, but these concepts are worth considering when developing interventions for children. If a child’s mental model of happiness and how it can be achieved does not match the model forwarded by a particular intervention, the intervention’s effect may be limited for that child. Researchers should make the effort to engage with the mental models of the particular child audience and, if necessary, work on changing counterproductive belief structures before deploying a positive technology intervention.

The children’s designs pointed to several specific features and engagement approaches that may increase the appeal of positive technologies. One noteworthy aspect of our findings is that participants often imagined technological solutions that could understand and react to various internal states, such as thoughts and emotions. Indeed, a growing number of efforts are attempting to glean psychological and emotional states from various affective computing technologies as diverse as electroencephalograms, galvanic skin response, and automated sentiment analysis on social media. Positive technologies use such features may have particular appeal for children, who are still learning to understand and interpret their affective states and the affective states of others. Another noteworthy aspect is in the number and diversity of approaches that the children posited for encouraging sustained engagement with interventions. While gamification and social interaction were two important approaches that have been considered in previous interventions (eg, [70,71]), there were also a few surprising ideas. One of these surprises was sensory engagement. Many of the children’s ideas posited that somebody could be motivated to engage with an intervention simply because it was beautiful and appealing to the senses, whether it be visual, aural, olfactory, or haptic. This is not a well-explored approach in the design of positive technologies, and it would be interesting to know the smells associated with happiness (our children suggested some, which included warm chocolate chip cookies and the smell of one’s own bed). Other surprising ideas were physical activity (as an engagement approach, not outcome) and humor.

Another design insight from this investigation emerged from observing the types of technologies that children cited in their inventions. It was clear that children were not drawn to interventions for laptops or desktops. At the very least, the implication of this is that Web-based interventions for children should be designed using a mobile-first paradigm. However, we should emphasize that this is just a temporary solution, as recent studies highlight that sustained engagement with such interventions is fairly minimal (eg, visits drop from an average of 5.19 per week during the first 2 weeks to 0.85 per week by 6 weeks later [72]). Indeed, there may be an opportunity to increase engagement by thinking outside the box (or the computer, as the case may be here). The children in our study suggested solutions that went beyond apps and websites, to consider several instantiations of embodied computing. These instantiations included wearable accessories and apparel, toys and gadgets that may operate independently or in conjunction with a phone app, smart furniture and home infrastructure, robots and drones, and public kiosks and displays. It may be fruitful for designers to consider their positive technology interventions not as sites that children visit, but rather as tools that live alongside with them in the real physical world.

### Limitations and Future Work

Our methodological approach has its limitations. Participatory design is an inherently subjective process that was likely influenced by the specific contexts, lenses, and biases of both the researchers and the children involved in the process. The same process carried out with another group of children or by other researchers may lead to a different perspective or emphasis in the findings. As such, we strongly encourage the replication of this work for greater confidence in the generalizability of these findings. Another limitation of our approach is that we started with three empirically supported happiness-increasing strategies (gratitude, mindfulness, and problem solving) rather than using a more general starting point such as *any* strategies that help make children happy. We believed it was more useful in our case to begin from such a starting point because it would help promote fidelity to the science of positive psychological interventions while still allowing some flexibility for the children to be creative and design new ideas. However, future work could use a different set of happiness-increasing strategies or work with children to generate novel happiness-increasing strategies within this age group. Finally, due to the constraints of the 8-week study, we were not able to develop any ideas into functional prototypes. Future investigations could develop functional interventions based on the underlying concepts expressed in the ideas created and vetted by our participants, could further iterate these prototypes with another group of codesigners, or could test such interventions through controlled in-the-wild deployments with children. We note that a full replication of our complete process could be challenging—not all research teams might be able to find community partners to conduct an 8-week program—but the principles of participatory design and cooperative inquiry can be applied in a shorter study. However, even though our process introduced challenges, we believe it offered insights that would not be possible in a shorter investigation.

Overall, this study is an important step forward in the design of positive technologies for children. It was truly an interdisciplinary undertaking, combining human-computer interaction and participatory design with positive psychology and positive psychological interventions. The dual focus of exploring design ideas while providing tangible benefits to the participants may be a useful approach to conducting research in school-based settings, where the first priority is returning value to the children. The results of this study reveal children’s understanding of three major concepts in positive psychological interventions (gratitude, mindfulness, and problem solving) and highlight strategies (such as directly engaging with thoughts and emotions, games, social support, and multisensory experiences) that might be critical in producing engaging interventions and technologies that would capture children’s interests. The future of positive psychology and positive technology would be well served by integrating more participatory methods and by listening to the voices of those they intend to support through interventions and technology.

## Appendix

Multimedia Appendix 1 Specific age-appropriate positive psychology topics and exercises used for instruction.

Multimedia Appendix 2 All ideas generated and expanded in the codesign workshops.

## Abbreviations

RQ: research question
Y.O.U.: Youth & Opportunity United
