# Supplementary material for: “Happiness Inventors”: Informing Positive Computing Technologies Through Participatory Design With Children
Source: J Med Internet Res. 2017 Jan 17;19(1):e14. doi: 10.2196/jmir.6822 (PMC5285607; doi:10.2196/jmir.6822)
Supplement: Multimedia Appendix 1 [file jmir_v19i1e14_app1.pdf]

Positive Psychology Topics and Specific Exercises Providing Instruction in Skills.

Gratitude	Mindfulness	Problem-Solving
**The Gratitude Song.** Students watch a YouTube video of a song describing and expressing gratitude.<br><br>**Looking for the Good.** Children write down 2 good things that could come from things that would generally be viewed as negative.<br>• Losing a sports game<br>• Experiencing a power outage<br>• Moving homes or schools<br>• Sharing something with someone (like a sibling or classmate).<br><br>**Writing "Super" Thank You Notes.** Children write a note to someone who has had a meaningful and positive impact on their lives. Children receive an example of a "Super" Thank You Note and are provided instructions to make the note specific, tell a story, and outline what the person did.<br><br>**Gratitude Mad Libs.** Children complete a Mad Libs style worksheet that constructs a note of gratitude to someone in their lives.	**Minds in Jars.** Children create a physical artifact to represent thoughts, emotions, and images in that might occur in one's mind by suspending glitter in a mason jar. We discuss the how these aspects can activate a student just like shaking or swirling the jar. Students then practice a breathing relaxation exercise while the contents in the jar settle.<br><br>**Drawing for "Focus."** Children pick an object in the classroom and spend 7 minutes drawing that object while focusing on details that they might overlook at first glance.<br><br>**Body Scan.** Children are led through a brief guided meditation to notice and focus on progressive parts of their body.<br><br>**Creating a "Positive" Image.** Children write down a goal or dream and led through a positive imagery exercise including a story about themselves achieving that goal including actions, emotions, and each of the 5 senses.<br><br>**Mind, Heart, and Body Messages.** Children are taught to distinguish different signals in their body by	**Sherlock Holmes vs. Merlock Worms.** Children listen to a story about two detectives Sherlock Holmes and Merlock Worms who have two different approaches to solving a problem. They then participated in a role-play illustrating another example of acting like Sherlock Holmes vs. Merlock Worms. Children then reflect on lessons learned from this story and the role-play.<br><br>**Chicken Little.** Children first recall and summarize and then read and reflect on the Chicken Little story. Children are asked to write about one time they acted like "Chicken Little" in their own life.<br><br>**Thought Comics.** Children are provided examples of comics that illustrate how thoughts impact people's actions and feelings. Children then created comics for examples in their own life when thoughts played a role in how they acted or felt.

	writing and discussing thoughts, emotions, and physical sensations.	
