# Supplementary material for: “Happiness Inventors”: Informing Positive Computing Technologies Through Participatory Design With Children
Source: J Med Internet Res. 2017 Jan 17;19(1):e14. doi: 10.2196/jmir.6822 (PMC5285607; doi:10.2196/jmir.6822)
Supplement: Multimedia Appendix 2 [file jmir_v19i1e14_app2.pdf]

Ideas from Brainstorming Workshops

(Note: these were written in shorthand during the ideation sessions and may require understanding the context of the session in order to interpret)

Gratitude Mad Libs App

Game that donates money

Makes letters you can send to people

Game that makes gratitude letter as you play

Game word search that the words are thankful words

Pick a celebrity and send gratitude to them

App that sends food to people who need it while you shop (by buying things on your to do list)

Searchable gratitude categorist that you can type or write

Click a song to cheer people on

Send stuff to multiple people - like Clash of Clans

Sends letter

App that changes bad posts to good posts like autocorrect (F M vote)

Gratitude - plays something to cheer you up

A survey app that takes you to a video based on how you feel (which are the survey questions)

Parents shop

Take videos about things you love about your parents

Pick the age of your parents and it will give you ideas about what people in that age range find funny (will make them laugh)

Pokemon Go gratitude app

App that you take pictures of people doing good things and it uploads to a map where people did them have to find people doing good things (like Pokemon Go)

Take pictures of people doing good things donates to charities

A game that each time you lose it donates money to charity

Youtube donates money to charities as people view the video (K vote FINAL CHOICE)

Work outs that donates money to charities when you do the work outs

Take pictures of your old clothes that don't fit and helps donate them to charities

Different activities within the app that when you do it donates it to charities

Motivational video each day

Makes photos look nicer

People tell the app sad things and it sends videos to cheer them up

Sends stuff to people who are sad

When bad things happens sends videos to people

Motivational each day - motivational message

Cat Videos (F M K vote FINAL CHOICE)

Care Bear

Shirt that can tell when you're feeling down

Laptop computer that has gratitude words on the outside

Drone that will drop candy when you do good things

Drone that will say nice things

Hat that plays music

Picture board at school - text good things to the board and it displays them

Stuffed animal

Pillow pets

Giraffes that have something good on them animal necklaces that display words

Shoes with different gratitude words that appear

Steps in your house that can read how sad you are - motivational quotes

Tablet that turns into a toaster

Toast with grateful words

Hat with kind words

Hat that knows what to play to make you laugh

Lemonade stand that donates to charity
Kindness -> charity lemonade stand when you turn in proof you did nice things it donates money to charity and you get free lemonade

Microwave that gives free food

Tv that only plays music that makes you happy

Stores with kind words

Electronic billboard in town that is a kindness leaderboard

Flowers

Trivia - tells you things - get something for rewards

Hololens - pictures and words that dare you to do kind things

Watch that shocks you when you say bad things

Camera takes pictures of kindness - makes something with them

Fundraiser with video games - based on winning

Toaster that gives you pop tarts for doing good things

Pepsi bottle caps with kind emojis or candies with kind emojis

App for making apps as gifts for others

Button you push when grateful to share inspirational message

Hotline to call when you feel bad

White board for leaving messages for friends

Inspirational music lip-synching

Free games let you thank the developers

App for drawings to give as thank yous

App that helps you write thank you notes

App for stabbing a friend you're mad at virtually

Phone blows up when you're not grateful

Phone encourages you to be grateful by giving more minutes

Turn your phone to talk to someone feeling sad

If you send a thank you note you get funny animal videos

If a person asks for help with feeling sad you call them

Gratefulness sensor

When you walk by somebody who is sad it lets you know

App to make you happy by letting you do things you find fun

Call your friends for you button

Facetime your friends button

Notebook that opens up if you're feeling sad

"Talk it out" app

Put your sad thought in a box

Beat up pretend person

Write a diary app

Show you funny videos when sad

App tells you to take a break

A pillow you can talk to

Take a walk app

Camera to see happy memories with somebody again
Hang out with family with an app
Music player
Something inside a pillow to talk to when sad
Pillow that plays music for you
Physical device to bring along
Play games on a computer or tablet for movies
Helps you sleep
Robot that knocks you out so that you can sleep
Sleeping gas
Take a break in a car
Something to help you play with pets
Ball throwing for your dog
Helps you get inspired
In the morning fixes hair and irons clothes
You have to punch something to get a treat
Keeps track of activities with a particular person
Idea robots of what to thank people for
Timer reminding you to thank someone
Help you take your time writing a note
A whiteboard to keep track of your thoughts
Squishy stressball
Device for when you're bored
Slapping device when you're not grateful
Play video game
Machine to take care of you
Keep track of how much you're reaching
Borrow a dog for a bit
Box with a present inside for somebody else
Pictures in your happiness box
Ball for remote sports
Keep notes of good things that happen
A basket with toys
Word translator
Math games
World baker
App that shows you how to cook
Zombie apocalypse escape game
App that find you new music
App that calls mom for you
Grocery store locator & price comparing
Shoe locator
Self-diagnosis doctor app
Baseball card trading app
Facetime and cooking interactive chef
Volume control
Sound amplifier
Hydration app

App that provides workout ideas
Racing game
Fries finder
Animal identifier
Word document app
Mail app
Emergency call app
Virtual clothes try-on
Train schedule updater
Best selfie locator app
Universal ruler app
Wheechair control app
Holographic watch
Basketball rebounder
Automated shoe rack
Extender arms
Gloves to climb walls
Outfit combiner
Real life tardis
Time machine
Thing traveler
Hover can
Hover boots
Hover-board Uber
Sea hummer submarine
Gas station at sea
Take my selfies floating phone
Holographic movie player
Pushup helper
Robot that shops for you
Shrink ray or growth ray
Watch transferor
Shoe size adjuster
Backpack that adjusts into a moped
Teleporter
Invisibility cloak
Self-drying clothes
Allergy (medicine?)
Mary Poppins style bag
Food regeneration
Personal ATM
Moveable walls
Digital mason jar (mind-in-a-jar)
Throw what you're thinking in a jar
Mind reading thought destroyer
Apps that reads your mind
Something that tells people to get out of the room
World volume control

World freezing app
[name omitted] disappearing app
[name omitted] disappearing app
Stop talking app
App that scans your body
App that puts you to sleep
Sister kicking stopper to go to sleep
Something to make someone sleep
Not having TV on
Music slowing
Robot grandma
Talk to mindful people
Sleep promoter
Sleep hoops for putting you to sleep
Energy promoter app
Mountain dew dispenser
Candy
Coffee
Sodas
Sugar
Peers
Pixie Stix
Punching bag
Candy world VR that gives real candy
Christmas VR - presents
X-Box helps prepare mind
PE to make more active
Pencil that writes for you
Pencil that shocks you when your head moves too fast
Watch that will expand when your mind races
Teleporter app to a spa
Teleporter to a mindful candy world
Massaging robot
Bed air freshener - smells just like bed
Milk sprayer
Cookie smeller
Cookie order app
Transport you somewhere relaxing
Backpack turns into a bed roll
Punching pillow that you can punch when you're mad
Baking glove that shoos people away
Mind clearing button
Glasses that see closer with auto zoom
Self-wrapping push-button blanket
On your ear reminding you to pay attention
Headphone that lets you know you're not paying attention
App for calm music
Door for letting thoughts in or shutting thoughts out

App for listing ideas for others to comment on
Alexa or Siri take this idea and let me forget until later
Math problems
App of top 10 mindful people and what they did that day
App that figures out where your body is tense
Massage suit for areas where you're stressed
App that draws as you body scan
Room painted in a calm color
Teleporter to the beach
Sound box backpack that blocks out all external sounds
Drawing app for when you're stressed
Math problems that are easy to do
Mind reader to help you figure out if you need something
Video game changes based on how you feel
Stress-killer punching bag
Which is realistic like boxing
If sad phone auto-shows cat photos
In a game cats replace all the characters
Phone camera captures facial expressions
Extra points on games if you've been happy all day
Brain-wave listening device to see how you're REALLY feeling
If tired Siri turns off all technology and yells at you to go to bed
Robot to spray water when you're tired
Brain-in-a-can represented by rocks in a can
App showing what you're thinking about
Meditation app for running
Senses how calm you are. If you're not calm you have to go to time out
A drawing book
A cartoon coloring book where characters start moving once you finish coloring them
Idea trashers
Mindstore that reads your mind and saves thoughts for later so you don't have to worry
Gopher game hit ideas that you want to get rid off
Shirt that encourages mindfulness with messages
Mindfulness couch
Dress that reads your emotions and blows up (like Marilyn Monroe) based on how calm you are
Propeller hat spins to show emotions
Hoverboard that floats depending on how calm you are
Shoes that glow depending on your thoughts
A wig that changes craziness based on your emotions
Lipstick that changes color based on your thoughts or emotions
Personalized activity booking to plan the activity to make you feel better
Ear buds with music like athletes before a game
Ear buds play the same music as a celebrity who was feeling similar emotions
Donut on your head that reads your mind
Shows you movie clips where actors were feeling the same emotion that you were
Another idea.
Relaxing music app
Music player that turns off when you're asleep

Robot that holds conversations with you
Antigravity machine
Coloring book app
All-encompassing ebook app
Comic book app
Teleporter
Shopping app that weeds out fake products
A robot that walks your dog for you
Robot cat or dog
Invisible phone case
App that uses your location to point out cool things nearby
App that sells furniture or beds
Machine that helps you with your homework
Phone case pillow
Phone case that heats up or cools down your phone
Body scan app
Tutoring app
Predicting app
Gadgets that refill your drinks for you
Phone case cup holders
Gadget that you wear that tells you if something is wrong in your body or if you need to go to the doctor
Recording app
Naptime app tells you how long to nap for
Advanced alarm won't let you sleep in
Transportation via cannon
Tracking app lets you know when guests will get there
Pen eraser
Robot that cleans the whole house
Hat that connects to phone and can receive messages
Robot that provides food
Chicago sports games
Mind-in-a-jar app
Video app for animals
Basketball knockout app
Volleyball app
App for online shopping for technology
App that suggests books to read
Stress relief app
Interactive stress ball when throwing it in the air it lands on the game you play
App that boosts adrenaline
Online notebook
Anger measuring app
Portable universal charger
Invisible purse or bag
App that can register other people's emotions
App tells you who you should hang out with
Pencil phone
App that lets you draw out your emotions

Create you own emojis
High five creator
Talk on your phone
A slapper - if you fall for something
Forgetter machine
Tricky homework
Positive it gives you something you want
Don't do it again
Distractor - go do some other activity
Game that helps you solve problems
Math games
Crazy alternative generator
Rainbow glasses or contact lenses
3D Glasses
Book
Wake up gadgets
Step retracer
Map to solve problems
Box that helps you think outside the box
Punch you in the face if you jump to conclusions
Mind miles per hour
Time watch
When someone takes stuff calls the police
Machine that gets to your friend to solve your problem
Sad movies
Happy movies
Top 10 problem solvers in state
Ask the experts - problem solver
Google Solve
Build a tower that breaks up your problem and it helps solve the problem
Throw thoughts into a bag
Machine to help you generate ideas
Student planners for students to not forget stuff
World pause button to help you stop and think
There's a lock on fun activities that won't unlock until you do homework
Journal that's shared with a friend so you can help rethink stuff
Phone senses when you're stressed and calls or texts a friend
"After Nap" button to pause a decision until after you've had a chance to nap
Magical eraser that fills in alternative ways of thinking about something
Magical eraser that only erases inaccurate stuff
Something that helps come up with 50 examples of you being smart
A clock that tells you when you forgot something
Fridge inventory keeper and shopping list printer
Something that lets you know how other people think about you
Something that draws your life as a comic book
Book that lets you write down bad and good times from your life but you can only see the good times when you look back on it
Something that gives counterexamples to bad thoughts (e.g. if you think ÒI'm dumbÓ it shows you all

the smart things you've done)
App that displays how many people in the world are going through the same situation as you (e.g. Ò1 million other kids forgot their homework todayÓ)

Translated Skype with a person who is facing the same problem as you to let you talk it out
If you're thinking about doing something stupid (e.g. jumping off a roof) an app shows how many people have died doing the same thing
App that shows other peoples reflections on a choice you're currently considering (e.g. did they regret it?)

Ice cream delivery machine

App for school and what to study in school

App to tell you to go to sleep

Something that gets rid of pain

Something that lets you make 16 wishes to change you life

A pizza delivery drone

Something you drink to take you back in time

Time travel machine to change bad decisions

Mom drone to remind you that you are loved

Other family members can also be drones

Text-a-friend hotline for when you feel down

A phone tracker for not forgetting stuff

An app when forgetting a meeting that will just remove it from your schedule

A bee robot for getting your bike back if it was stolen

A bully defender

Something that helps you think about and envision your future

An "it gets better" crystal ball to show something good from your future

Tissue papers with answer to the test

Door lock that tells you what you forgot

Paper clip for you notebook that tells you what you forgot

Fast skateboard for not missing your flight at the airport

App with problem solving skills game

Solution generating app so you consider multiple alternatives

Type in a problem and it prints possible solutions

Game that teaches you how to make better decisions

Machine that fixes broken things

All-purpose super glue

Clue generator

Question generating app

App with tutorials for how to make stuff

Mystery game

Riddles app

Treasure hunt like Pokemon Go

Scanner gadget to match people with names

Lie detector

Liar detector

Fill in the blanks pen

Fact checking app

Recording watch

Idea storing glasses

Switch that always lets you win

App that reminds you of your own past actions
App that helps you w or homework
App where you make decisions and choices and get prizes for correct choices
App that gives you tricks to make people forgive you
Toy building game
YouTube how to videos filter
Virtual mom
App gives you solutions and you have to identify problems
App that identifies positive outcomes
Solution generating headband
Take a picture and app will show you how to make things better
Life's most common problems to best solutions app

Ideas Kids Chose to Write and Draw in their Invention Notebooks

Shows a panda-like robot with red glowing eyes, carrying a bat that says “Good Night!” If you’re having a bad day, it will knock you out with the bat so you can go to sleep, end your bad day, and start over again the next day. –P1

Shows drawing of a grey bed with P1 asleep in it and a yellow device with green lights and red music notes coming out of it. The device plugs into a wall and plays music until you are 100% asleep, so that your thoughts don’t keep you up. –P1

Shows a panda-like robot with red glowing eyes, holding a piece of paper with a completed homework assignment. Opposite page reveals alternative designs for face and body and shows the face with the casing removed. –P1

Shows a display screen with the video of your friends playing soccer together, but also give you a video game controller that can be used to control a physical soccer ball, so that a person playing a video game can play with somebody playing soccer in real life. –P2

Written out description: “an app that will scan [a thank you letter] and if you send a negative message they will replace it with a thanking message.” –P2

Written out description: “when you send something thankful, a robot would do some chores for you.” –P2

Written out description: “a robot that will read out your message to you before you send your message.” –P2

Written out description: “I think that one invention that can help most kids practice mindfulness is a tutor app so that you can practice every day and get good grades.” Drawing shows a tablet or phone with colorful text to select the person’s grade-level. The second screen says “Ready, set, go!” and has a series of math problems on the screen. –P2

Drawings on a set of iPhone paper templates describe “Dear Diary The App!” The first screen has you select whether you are feeling sad, happy, or mad. If you’re sad, the app lets you write your entry and you can share it with a friend via text or you can call your friend if you didn’t have a good day. If you’re mad, it shows you a game where you can punch a pillow with a picture of somebody you are mad at. –P2

Shows a drawing of a robot with treads, two extendable arms, and a screen and camera on a long neck. Above it, shows squiggles representing a thank you note. An arrow points to the part of the note that is identified as being “negative.” The robot changes this part to be “positive.” The opposite page shows the robot sketched out in more detail and named “CB1.” It says that the robot will cost $112 (corrected to $112.99) and will be available in white or gray. It describes that you can send a message to a specific household’s robot by entering the 4-digit number on the robot’s back. –P3

Written out description: “I think that the one invention that can most help kids practice mindfulness in an idea saver, because say you have a good idea but you don’t need it now, but you will later…” A drawing shows a black-and-brown ear bud that “goes into ear, reaches brain to save idea.” Another description amends that it “listens to your ideas and saves them for later” and shows a drawing of a person’s head wearing the ear bud. –P3

Written out description: “Final gadget ‘Personal Assistant Robot’ (P. A. R.) helps with things that stress you out. It is a robot that helps with homework, chores, holds conversations with you, helps you with homework, cleans for you, keeps reminders, has a scanner, connects to a free app for on-the-go, it is Yoda-sized, it can be programmed to make breakfast, has an emergency shut-down button, it’s huggable, it can call 911 and send them a video record of what’s going on, it has USB and outlet ports on its arms.” The technical parts of the robot are described in a callout “computer base, remote controlled via app (password protected), video camera, speech box, arms w/ ‘hands’, powered via battery (like a car battery).” It also clarifies that the app is free but the robot costs $899. –P3

Drawings on a set of iPhone paper templates describe an app named “Read My Recipe.” The first screen has a “Start” and “Help” button. The second screen allows you to take a photo as you complete the step: “1/3 cup of sugar” and shows an image of sugar being poured into a mixing bowl. The last page shows the recipe being translated into another language to be shared with others. –P3

Shows a drawing of a robot with treads, a punch bag body, a screen with a face, and a display above the face showing the number of points. A hand in a boxing glove is reaching out to punch the robot. As it punches the screen with the face appears to develop cracks and the number of points increases. –P4

Written out description: “I think that the one invention that can most help kids practice mindfulness is a music player that turns of when you are asleep, because that will help you sleep.” A drawing shows a child in bed with a speaker above them producing red music notes. –P4

Shows a drawing of a robot with treads, two arms, and a smiling face. It is holding a pencil. In front of it is a page labeled “homework” with a lot of writing on it. The robot is surrounded by purple question marks. –P4

Written out description: “One ant [written “ant,” but meant “bee”] that protects your stuff.” Shows a person with horns approaching a bike saying “I’m going to steal this bike. Hahaha!” A small bee-like robot drone says “No, you’re not, I’m going to poke you!” Image below also shows four bee-like drones combining forces to deliver a pizza. –P4

Drawings on a set of iPhone paper templates describe an app without a name. The app shows a person who is mean to you and lets you take out your anger on them. The first screen shows throwing stars thrown at the figure, with a narrator saying “finish him.” The second screen shows the figure saying “no” while a hand prepares to stab it with a pencil. The third screen shows the figure saying “I will kill you” while a hand prepares to hit it with a hammer. –P4

Drawing on a set of Android phone paper templates describe an app without a name. The first screen shows two people talking over each other. They are circled and crossed out. The second screen shows one person highlighted and talking, while the other person is circled and crossed out. –P4

A post-it note drawing on the page is described as a “gel music pillow” and shows a fluffy pillow with a music speaker built into it. –P5

Written description: “If you say something nice, a drone drops candy on you. The more bad things you say the more the shock you get.” The drawing shows a spherical robot with a displayed smiling face, a propeller, and a claw below it. Another drawing below shows a watch that has a smiling face on it and displays a “Nice meter: 4/5” and a “Bad Meter 3/5.” –P6

A drawing shows a watch displaying a bar meter, the number 25, and an up arrow. The number is described as “current points.” The arrow is described as “scroll up for ideas about how to get points.” A smiling face above the image identifies this as an idea that rewards you for practicing gratitude. –P6

Written description: “I think that the one invention that can most help kids practice mindfulness is an ‘Idea Trasher,’ so if you have a bad idea or think of something scary you can put it inside there to forget it.” The drawing shows a face with vampire teeth with speech bubble “Another one!” and eyes that are slots for inserting the idea. –P6

A drawing of an iPhone shows a piece of paper that says on it: “I will fail.” There are scissors on the screen for cutting up and destroying the paper. A message on the screen displays “FATALITY” in red once the bad idea is destroyed. –P6

Written description: “Robots/apps that will help you with homework, shopping, and writing.” –P6

Written description: “Make the world calmer. A backpack with a sound proof sound box around it, calm music, and a smell dispenser that creates a cookie smell.” Off to the side also says, “teleporters.” [ostensibly to avoid the commute] –P6
Written description: If you have a “stuck feeling,” what can help is an “idea trasher or a mind clear button.” –P6

Written description: To help you “pay attention” to you body messages, you can have a “massage suit that records your thought signals and does a body scan” to massage parts of you that are tense. –P6

Written description: “We wanted to make the world calmer by making a device that has three parts. The earpiece blocks out any noise. The backpack gives a massage and has a fan. And there is a good smell dispenser that shoots out. Me and my fellow inventors think this will work because it can make the unpleasant things in life more pleasant.” Opposite page shows three numbered sketches: an ear with a bud inside it, a face smiling at good smell wafting from the earpiece, and a backpack. –P6

A drawing shows a box with a video display inside. The display has videochat with a person labeled at “Random Kid” and the child is saying “LOL.” The drawing is labeled as “Think Outside DA Box!” The written description below describes: “When you open a box, shows people who help think through you choices on Skype. You can either show your face or create an avatar.” It also states the price of the device as $299.99 and states that use of it will require “wifi and a Gmail, Instagram, or Facebook account.” Line below says “sponsored by Skype.” –P6

Written description: “I would create a littleBit that has rainbow lights.” –P7

A drawing shows an arm wearing a red smart watch, which has a labeled speaker on one side and four function buttons. The buttons correspond with a “health checkup,” a “homework helper,” a watch showing the time, and one unlabeled button. There is also a drawing of an iPhone that can connect to the watch. The description names the invention “RW positive (RW+ for short)” and states that it is “to help take care of yourself.” –P8

Written description: “I think that the one invention that can most help kids practice mindfulness is a mind trasher, because it can get things out of their heads.” A drawing shows an iPhone with a written label “DJ Khaled” and a trash can labeled as “M.” The written label can be dragged into the trash can to trash it. –P8

A drawing shows an iPhone screen labeled “TrezHunt.” The introduction screen states: “A place a time, a way for you, a place where you can, go and eat popcorn too!” A button at the bottom of the screen lets you get hints and shows that you still have 6 hints left. The next phone screen shows the person taking a photo of a movie theater and says “you found it!” –P8

A drawing shows an iPhone screen with an app named “The Life Detector.” There is a button labeled “Scan!” and there is a place to “pick what your category is!” [Lana’s edit: I think this was actually the “Lie” detector which was supposed to let you scan yourself or others for inaccurate thoughts about yourself] –P8
Written description: “I think that the one invention that can most help kids practice mindfulness is the ‘freeze app,’ because it can make everyone freeze.” The drawing shows a red device with two orange screens and a pink wearable bracelet device with a darker pink and a purple button. There is a drawing of a girl frozen in place. [Lana’s edit: this is an app that lets you stop time for everybody but you to give you a moment to reflect.] –P9

A drawing shows a box labeled as “The Box” with a description below reading “dun dun dunn.” As the box is opened, a hand labeled as “slappy hand” comes out of the box. A girl in the drawing says “oh my!” [Lana’s edit: this is a device that will slap you if you’re having dumb thoughts about yourself like “I will fail.”] –P9

A drawing shows an arm wearing a watch, labeled “Buttons on watch controls drone.” Four buttons on watch are labeled: “fast,” “slow,” “high,” “low.” The drawing next to it shows a spherical drone opening a claw to drop pieces of candy. –P10

Written description: “A watch that measures how grateful and positive you’ve been. And a kiosk where you can cash in your points for candy, movie tickets, Legos, and water bottles.” Drawing on next page shows a blue and grey rectangular kiosk with prizes behind glass. Several parts are labeled: “prizes,” “claw” for getting the prize, “key pad for choosing your prize,” and a hole for “insert your hand here” to get the point information off your watch. –P10

Written description: “I think the one invention that can most help kids practice mindfulness is something that shoos your sister/brother away when you are baking, because it would make me more relaxed.” Drawing below shows a green and black device with a single button. It is labeled “Makes a disgusting smell that makes your sis/brother get away.” –P10

A drawing shows a girl confused by a math problem. There is a computer with a keyboard and mouse that helps her figure out the answer. It is labeled “Help with H.W. or Something Else.” –P10

A drawing labeled “Scanner” shows a device that scans three kids in a row. Getting to the last kid, it glows and beeps. There is also a drawing of an eye. [Lana’s edit: I think this is a lie detector] –P10

Written description: “I think that our idea can help kids practice problem solving, because the positive thoughts come to the front.” The drawing shows a tablet app with fish on it. Each fish corresponds to a thought. The positive ones come to the foreground (“I am good at math”) but the negative ones swim to the background (“I am dumb”). –P10

Drawings on a set of iPhone paper templates describe an app named “Catatude.” The first screen shows a drawing of a cat. The second screen shows YouTube where you can search for videos to watch and has a drawing of a video about football. The last screen shows a video of a cat on YouTube. There is a part that shows how much money your view of that video helped generate. The money is donated to charity. [Lana’s edit: the idea was that you feel better by watching funny videos you enjoy and the money your views generate go to charity which helps you feel even better and helps out others.] –P10

There is a drawing of a black, green, and red drone with a propeller approaching a smiling person. –P11

There is a drawing of a humanoid robot with a glowing blue power core picking up something off the floor. –P11

There is a drawing of a box filled with many tablets. Each tablet can connect you to a different expert by Skype to help you make decisions in your life. –P11

A written description says: “Help take care of yourself” followed below with the like “homework.” There is a drawing of a printer/scanner-like device. You put your homework in it and it comes out done. –P12

Written description says: “I think that the category of ideas that can most help kids practice gratitude is helping others because it sets examples.” A drawing below shows a drone that rewards kids for helping others. –P12

A drawing shows a smart watch on a wrist. It has five colorful buttons and a screen that reads “reminder” and shows a reminder. Attached to the side of the watch is a green speaker. –P12

A paper iPhone template shows one screen of an app. The app is labeled “My Little Pony” and it has an image of a house, lake, and grass on it. –P12

Ideas Selected by Teams to Document as Videos

Gratitude 1: A watch that tracks how grateful and positive you are and gives you ideas to get more “gratitude points.” You can scan the watch at a kiosk to get a prize for your points, like candy, Legos, and videos.

Gratitude 2: A watch and app that helps you not stress out about forgetting things. It has a speaker and can display messages to remind you to do things that are good for your health and to do your homework.

Gratitude 3: A care robot that you can send thank you notes to. It helps correct any negative thoughts/ words into your thank you notes, to help improve your thank you notes. It can also help do chores for the person you are thanking.

Mindfulness 1: A Yoda-sized personal assistant robot that can do you homework, remind you of stuff, and make breakfast. It can also have conversations with you and it’s huggable. In an emergency, it can send you video to the police. It can also be controlled by a password-protected app on your phone.

Mindfulness 2: A backpack that has a “world volume” earpiece, a good smell dispenser, a fan, and a massage feature to make the unpleasant things in life more pleasant.

Problem Solving 1: A box with a tablet inside it that can help you videochat with experts so that you can carefully consider decisions and choices when you are making them.

Problem Solving 2: A tablet app that scans your thoughts and displays them as an aquarium on the tablet. The fish representing good thoughts swim to the foreground, while the fish representing bad thoughts slowly fade into the background.

Problem Solving 3: A lie detector device that can help you figure out if somebody has inaccurate thoughts about themselves or if they are lying about something.
